# Correction: Yu et al. HPV16 and HPV18 Genome Structure, Expression, and Post-Transcriptional Regulation. *Int. J. Mol. Sci.* 2022, *23*, 4943

**DOI:** 10.3390/ijms23147903

**Published:** 2022-07-18

**Authors:** Lulu Yu, Vladimir Majerciak, Zhi-Ming Zheng

**Affiliations:** Tumor Virus RNA Biology Section, HIV Dynamics and Replication Program, Center for Cancer Research, National Cancer Institute, National Institutes of Health, Frederick, MD 21702, USA; lulu.yu@nih.gov (L.Y.); majerciv@mail.nih.gov (V.M.)

In the published review [1], the HPV16 late promoter P_L_ in Figure 1a was wrongly labeled in the E6 open reading frame. The corrected Figure 1a below shows the HPV16 P_L_ promoter in the E7 open reading frame.

The authors apologize for any inconvenience caused and state that the scientific conclusions are unaffected. This correction was approved by the Academic Editor. The original publication has also been updated.

## Figures and Tables

**Figure 1:**
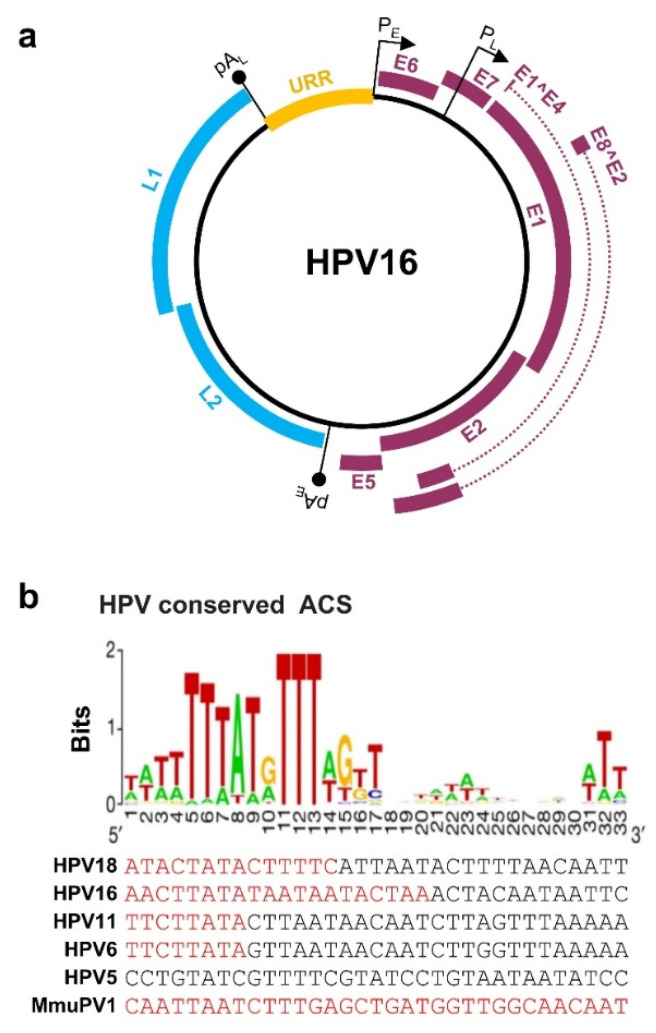
Papillomavirus genome structure, annotated ORFs, and conserved ACS in the origin of replication (Ori). (**a**) HPV16 genome and annotated ORFs. The non-structural proteins from the early region of the genome, including E1, E2, E1^E4, E5, E6, E7, and E8^E2, are shown in purple. The viral capsid proteins, L1 and L2, from the late region of the genome are shown in blue. URR (yellow), upstream regulatory region. P_E_ and P_L_ mark the early and late promoters, and pA_E_ and pA_L_ stand for the early and late polyadenylation sites. (**b**) Conservation of the mammalian ARS consensus sequence (ACS) [16] in the Ori of selected papillomavirus genomes. Shown in this panel are conserved bases with position weight matrix (sequence logo bits) of the 167 predicted ACS elements [16] in comparison with the viral Ori sequences from the selected viral URR tail (red)-head (black) regions. Adapted with permission from Ref. [16]. Copyright 2018 Springer Nature. ARS—autonomously replicating sequence.
